# Three-Dimensional Partial Discharge Localization Method Based on Hybrid RSSI Fingerprint Maps and Minimal Deviation Model

**DOI:** 10.3390/s26216962

**Published:** 2026-11-04

**Authors:** Can Zhang, Xingzhou Yan, Lingen Luo, Gehao Sheng

**Affiliations:** State Key Laboratory of High-Efficiency Special Cable Technology, School of Electrical Engineering, Shanghai Jiao Tong University, Shanghai 200240, China

**Keywords:** partial discharge, three-dimensional localization, RSSI, fingerprint, Kriging

## Abstract

The UHF received signal strength indicator (RSSI) fingerprint map method was recently proposed to overcome the restrictions of the time delay algorithm widely used for partial discharge (PD) localization. However, existing studies focus on two-dimensional PD localization, while many situations require three-dimensional information. In addition, PD localization accuracy is highly dependent on the precision of prebuilt fingerprint maps, which remains challenging to achieve. Therefore, this paper proposes a hybrid three-dimensional PD localization method that combines RSSI fingerprint maps and a range-based algorithm. First, limited test points on several planes were selected to build RSSI fingerprint maps. Then, single-plane smooth interpolation was employed for each plane to obtain smooth three-dimensional fingerprint maps. Here, the limited test points compensate for the non-line-of-sight (NLOS) effect, while the interpolation method reduced the workload required to build the fingerprint map. Thirdly, enhanced four-dimensional fingerprint maps were established based on the Kriging method. Finally, a minimal deviation model was proposed to locate the position of the PD source by analyzing the relative ratio of the RSSI values across different sensors. Laboratory validation shows that the average error of the proposed method is 0.69 m, which demonstrates its accuracy and practical application value of the proposed method.

## 1. Introduction

Partial discharge (PD) reduces the insulation performance of electrical equipment. After a period of time, electrical equipment may fail, ultimately causing power outages. It is therefore necessary to detect and locate the PD in electrical equipment to ensure the stable operation of the system [1,2,3]. Due to its excellent anti-interference, stability, sensitivity, and propagation speed, UHF is one of the most effective methods for PD detection and localization [4,5].

Commonly used methods for PD localization are predominantly based on the time difference of arrival (TDOA) [6,7] or angle of arrival (AOA) [8,9]. The TDOA method determines PD localization based on the time difference in the signal. It requires a high-speed synchronous sampling system, which is costly in terms of hardware [10]. In non-line-of-sight localization, the accuracy of the AOA method is affected, so this method is not suitable for PD localization in substations [11].

In recent years, scholars have tried to use the received signal strength indicator (RSSI) to locate PD [12,13,14,15,16]. Methods using RSSI can be divided into two types. One is based on signal attenuation models, and the other is based on the scenario analysis of RSSI fingerprint maps. It should be noted that the electromagnetic and spatial environments of substations are very complex. Signal attenuation models can be significantly affected in a non-line-of-sight (NLOS) situation, making it difficult to achieve precise results [17]. The RSSI fingerprint map method is hardly affected by the spatial and magnetic environments, and has been shown to be more reliable [18]. However, existing studies on the RSSI fingerprint map method have focused on two-dimensional PD localization. In industrial practice, it is necessary to achieve three-dimensional PD localization to meet the needs of electrical equipment operation and maintenance. These methods do not actually achieve two-dimensional localization by generating fingerprint maps but directly predict the position based on the correspondence between known positions and numerical values. On the other hand, PD localization accuracy is highly dependent on the precision of prebuilt fingerprint maps, which is always difficult to obtain, especially for large regions. Therefore, a hybrid PD localization method that combines RSSI fingerprint maps and a minimal deviation model is proposed. The RSSI fingerprint maps must be built based on limited test points, according to the actual field conditions, to overcome the influence of the NLOS effect. Geostatistical interpolation is also used to improve the accuracy of the method.

In this paper, a novel three-dimensional PD localization method was proposed based on RSSI fingerprint maps, which are enhanced by employing single-plane smooth interpolation and the Kriging method. In the offline stage, a few three-dimensional test points on several planes are selected from the test space. A standard PD simulator is used to discharge at each point, and the corresponding RSSI value and coordinate were recorded. A single-plane smooth interpolation method was first employed to form a smooth three-dimensional fingerprint map for each plane. Then, enhanced four-dimensional fingerprint maps are established for the three-dimensional test points using the three-dimensional maps and the Kriging method. In the online stage, the RSSI values were recorded by different sensors when PD occurred. The position of the PD was determined by analyzing the relationship between the recorded RSSI values and the established four-dimensional fingerprint maps in the offline stage. Experimental tests are then performed to verify the accuracy of the proposed method.

## 2. The Hybrid PD Localization Method

### 2.1. Offline Stage: Establishing the Fingerprint Maps

#### 2.1.1. Acquiring the RSSI Values

It is impractical to perform a discharge test at every point in a substation. Thus, selecting several points to collect data is more feasible. Assume that the location of the standard PD power supply used for testing is *P_m_*, and the RSSI value received by sensor *S_n_* in the *k*th test is denoted by *r_n,m_*(*k*). The testing matrix is shown in Equation (1).

(1) $$R=\left[\begin{array}{c} \begin{array}{c} \begin{array}{c} \begin{array}{cc} r_{1,1} & \ldots \;r_{1,m} \end{array}\begin{array}{cc} \;\ldots & r_{1,M} \end{array} \end{array}\\ \vdots \end{array}\\ \begin{array}{cc} r_{n,1} & \ldots \;r_{n,m} \end{array}\begin{array}{cc} \;\ldots & r_{n,M} \end{array}\\ \begin{array}{c} \vdots \\ \begin{array}{cc} r_{N,1} & \ldots \;r_{N,m} \end{array}\begin{array}{cc} \;\ldots & r_{N,M} \end{array} \end{array} \end{array}\right]$$

(2) $$r_{n,m}=\frac{1}{L}\sum_{k=1}^{L}r_{n,m}\left(k\right)$$

where *N* is the number of sensors, *M* is the number of test points, and *L* is the number of tests at each point. ***R_n_*** = [*r_n_*_,1_… *r_n_*_,_*_m_* …*r_n,M_*] represent the RSSI values recorded by sensor *n* at *M* test points. Using the RSSI values recorded by the sensors, a fingerprint for each sensor can be established.

As the field noise and spatial environment might affect the measurement data, Gaussian filtering can be used to suppress the impact [19,20]. After filtering, the mean of the peak values of several measurement waveforms can be taken as the test point RSSI value. However, the intensity may vary when an actual PD occurs. To overcome the impact of the intensity, the RSSI value of each test point is normalized. The normalization operation for test point m is defined as

(3) $$r_{n,m}^{\prime }=\frac{r_{n,m}}{\frac{1}{N}\sum_{n=1}^{N}r_{n,m}}\;$$

#### 2.1.2. Single-Plane Smooth Interpolation Method

In order to achieve three-dimensional localization, test points should be selected from multiple two-dimensional planes instead of a single two-dimensional plane. In addition, on each two-dimensional plane, the number of selected test points is limited. It is also infeasible to perform the discharge test at every point on a two-dimensional plane. Thus, biharmonic spline interpolation is employed to obtain RSSI values at other points using a limited set of test points.

In the 19th century, draftsmen used to add weights to elastic beams or splines and adjust the size and position of the weights so that the spline shape could pass through each given data point [21]. This method was used to solve the problem of drawing a smooth curve. In a one-dimensional case, the displacement function of the elastic spline, when subjected to a vertical concentrated force δ(*x*−*x*’) located at point *X*’, satisfies the following biharmonic equation [22]:

(4) $$\frac{d^{4}\varphi }{dx^{4}}=6\Delta (x-x^{\prime })$$

where *φ* is the perpendicular displacement of the spline and *δ* is the delta function. The solution to Equation (4) is

(5) *φ* = |*x* − *x*′|^3^

If the Green function is used for interpolation with *K* data points *w_i_*, which are located at *x_i_*, the biharmonic equation to be solved becomes

(6) $$\frac{d^{4}w}{dx^{4}}=\sum_{j=1}^{K}6a_{j}\Delta (x-x_{j})$$

(7) *w*(*x_i_*) = *w_i_*, *i* = 1, 2, …, *K*

where *a_j_* is the weighting coefficient, which indicates the magnitude of the added force. *w*(*x_i_*) represents the displacement at *x_i_*. The solution to Equations (6) and (7) is a linear combination of the Green’s functions [23] of the added force at each data point.

(8) $$w\left(x\right)=\sum_{j=1}^{K}a_{j}{\left|x-x_{j}\right|}^{3}$$

The magnitude of the added force at each point can be calculated by solving the following linear equation:

(9) $$w_{i}=\sum_{j=1}^{K}a_{j}{\left|x_{i}-x_{j}\right|}^{3}$$

Once *a_j_* is determined, the interpolation for any point can be obtained using Equation (8).

Biharmonic spline interpolation in two dimensions is similar to that in one-dimensional cases. The interpolation function *w*(*x*) passing through *K* data points *w_i_* satisfies the following equations:

(10) $$\nabla^{4}w\left(x\right)=\sum_{j=1}^{K}a_{j}\Delta (x-x_{j})$$

(11) w(*x_i_*) = *w_i_*, *i* = 1, 2, …, *K*

where ∇^4^ is the biharmonic operator, and ***x*** = (*x*_1_*,x*_2_*,…,x_K_*) is the coordinate vector of the *K* data points in the two-dimensional space. The solution to Equations (9) and (10) is

(12) $$w\left(x\right)=\sum_{j=1}^{K}a_{j}{\left(x-x_{j}\right)}^{2}\left[\ln\left(x-x_{j}\right)-1\right]$$

*a_j_* can be obtained by solving linear Equation (13). The interpolation for any coordinate can then be calculated using Equation (12):

(13) $$w_{i}=\sum_{j=1}^{K}a_{j}{\left(x_{i}-x_{j}\right)}^{2}\left[\ln\left(x_{i}-x_{j}\right)-1\right]$$

By employing biharmonic spline interpolation, additional RSSI values at other points on a plane can be obtained using just a few test points.

#### 2.1.3. Method for Establishing Enhanced Four-Dimensional Fingerprint Maps

To achieve three-dimensional localization, a four-dimensional fingerprint map is required. In other words, the RSSI values of points across multiple dense planes are needed. A small number of planes is not sufficient; however, it is impossible to perform discharging tests on dense planes. Thus, the Kriging method was employed to establish a four-dimensional fingerprint map using the points of a few planes. The Kriging method combines traditional geoscience methods with statistical methods to form a complete formula system and can be used to study the spatial distribution of natural phenomena [24,25]. The Kriging equation is defined as

(14) $$\hat{p_{0}}=\sum_{i=1}^{g}w_{i}p_{i}$$

where $\hat{p_{0}}$ is the estimated value of point (*x*_0_, *y*_0_, *z*_0_) in a three-dimensional space. This value is a linear combination of the observed values *p_i_* of *g* points and the weight coefficient *w_i_* relative to the point (*x*_0_, *y*_0_, *z*_0_). To ensure an unbiased estimation in Equation (15), the weight coefficient should meet the conditions defined in Equation (16) [26,27,28].

(15) $$E\left(\hat{p_{0}}-p_{0}\right)=0$$

(16) $$\sum_{i=1}^{g}w_{i}=1$$

where *p*_0_ is the actual value of point (*x*_0_, *y*_0_, *z*_0_).

The Kriging variance, or Kriging error, of point (*x*_0_, *y*_0_, *z*_0_) is shown in Equation (17).

(17) $$\begin{array}{c} \;\mathrm{var}\left(\hat{p_{0}}-p_{0}\right)=\mathrm{var}\left(\hat{p_{0}}\right)+\mathrm{var}\left(p_{0}\right)-2\mathrm{cov}(\hat{p_{0}},p_{0})\\ =\mathrm{cov}\left(\sum_{i=1}^{g}w_{i}p_{i},\sum_{i=1}^{g}w_{i}p_{i}\right)+\mathrm{cov}\left(p_{0},p_{0}\right)-2\mathrm{cov}\left(\sum_{i=1}^{g}w_{i}p_{i},p_{0}\right)\\ =\sum_{i=1}^{g}\sum_{j=1}^{g}w_{i}w_{j}\mathrm{cov}(p_{i},p_{j})+\mathrm{cov}\left(p_{0},p_{0}\right)-2\sum_{i=1}^{g}w_{i}\mathrm{cov}(p_{i},p_{0}) \end{array}$$

The semivariance function is defined as *v_ij_*, as shown in Equation (18), which is equivalent to Equation (19).

(18) $$v_{\mathrm{ij}}=\frac{1}{2}E\left[{\left(p_{i}-p_{j}\right)}^{2}\right]$$

(19) *v_ij_* = *σ*^2^ − *cov*(*p_i_*,*p_j_*)

where σ^2^ is the variance. By bringing Equation (19) into Equation (17), Equation (20) can be obtained.

(20) $$\begin{array}{c} \mathrm{var}\left(\hat{p_{0}}-p_{0}\right)=\sum_{i=1}^{g}\sum_{j=1}^{g}w_{i}w_{j}(\sigma^{2}-v_{\mathrm{ij}})+(\sigma^{2}-v_{00})-2\sum_{i=1}^{g}w_{i}(\sigma^{2}-v_{i0})\\ =\sum_{i=1}^{g}\sum_{j=1}^{g}w_{i}w_{j}\sigma^{2}-\sum_{i=1}^{g}\sum_{j=1}^{g}w_{i}w_{j}v_{\mathrm{ij}}+\sigma^{2}-v_{00}-2\sum_{i=1}^{g}w_{i}\sigma^{2}+2\sum_{i=1}^{g}w_{i}v_{i0}\\ =\sigma^{2}-\sum_{i=1}^{g}\sum_{j=1}^{g}w_{i}w_{j}v_{\mathrm{ij}}+\sigma^{2}-v_{00}-2\sigma^{2}+2\sum_{i=1}^{g}w_{i}v_{i0}\\ =2\sum_{i=1}^{g}w_{i}v_{i0}-\sum_{i=1}^{g}\sum_{j=1}^{g}w_{i}w_{j}v_{\mathrm{ij}}-v_{00} \end{array}$$

The model for minimizing the Kriging error at point (*x*_0_, *y*_0_, *z*_0_) can be formulated as

(21) $$\left\{\begin{array}{c} \min f\left(w_{1},w_{2},\ldots ,w_{g}\right)=2\sum_{i=1}^{g}w_{i}v_{i0}-\sum_{i=1}^{g}\sum_{j=1}^{g}w_{i}w_{j}v_{\mathrm{ij}}-v_{00}\\ s.t.\sum_{i=1}^{g}w_{i}=1 \end{array}\right.$$

In order to obtain a set of *w_i_* to achieve the goal of minimizing the Kriging error, the Lagrange function *J* can be constructed to solve the problem.

(22) $$J=f\left(w_{1},w_{2},\ldots ,w_{g}\right)+2\gamma (\sum_{i=1}^{g}w_{i}-1)$$

We can then take the partial derivatives with respect to (*w*_1_, *w*_2_, …, *w_g_*, *γ*) and set the values to zero so that the optimal value of *w_i_* can be obtained.

(23) $$\left\{\begin{array}{c} \frac{\partial (2\sum_{i=1}^{g}w_{i}v_{i0}-\sum_{i=1}^{g}\sum_{j=1}^{g}w_{i}w_{j}v_{\mathrm{ij}}-v_{00}+2\gamma (\sum_{i=1}^{g}w_{i}-1))}{\partial w_{k}}=0,k=1,2,3,\ldots ,g\\ \frac{\partial (2\sum_{i=1}^{g}w_{i}v_{i0}-\sum_{i=1}^{g}\sum_{j=1}^{g}w_{i}w_{j}v_{\mathrm{ij}}-v_{00}+2\gamma (\sum_{i=1}^{g}w_{i}-1))}{\partial \gamma }=0 \end{array}\right.$$

After simplification, Equation (23) can be transformed into Equation (24).

(24) $$\left\{\begin{array}{c} v_{k0}-\sum_{i=1}^{g}w_{i}v_{\mathrm{ki}}+\gamma =0,k=1,2,3,\ldots ,g\\ \sum_{i=1}^{g}w_{i}=1 \end{array}\right.$$

This equation set for calculating the weight coefficients of *w_i_* can be converted to matrix form, shown as follows:

(25) $$\left[\begin{array}{ccc} \begin{array}{ccc} v_{11} & v_{12} & \ldots \\ v_{21} & v_{22} & \ldots \\ \vdots & \vdots & \vdots \end{array} & \begin{array}{c} v_{1g}\\ v_{2g}\\ \vdots \end{array} & \begin{array}{c} 1\\ 1\\ \vdots \end{array}\\ \begin{array}{ccc} v_{g1} & v_{g2} & \ldots \end{array} & v_{\mathrm{gg}} & 1\\ \begin{array}{ccc} 1 & 1 & \ldots \end{array} & 1 & 0 \end{array}\right]\left[\begin{array}{c} w_{1}\\ w_{2}\\ \begin{array}{c} \vdots \\ w_{g}\\ -\gamma \end{array} \end{array}\right]=\left[\begin{array}{c} v_{10}\\ v_{20}\\ \begin{array}{c} \vdots \\ v_{g0}\\ 1 \end{array} \end{array}\right]$$

where the vector $w=\left[\begin{array}{c} w_{1}\\ w_{2}\\ \begin{array}{c} \vdots \\ w_{g} \end{array} \end{array}\right]$ contains the weight coefficients used to calculate the predicted value.

To summarize, the value-estimating process for the three-dimensional point (*x*_0_, *y*_0_, *z*_0_) consists of six steps.

Step 1: Calculate the distance and semivariance of each pair of known points.

Step 2: Find a curve to fit the relationship between the distance and the semivariance so that the semivariance can be calculated based on any distance value.

Step 3: Calculate all the semivariances between the known points.

Step 4: Calculate the semivariances of the three-dimensional point to be estimated relative to the known points.

Step 5: Calculate the weight coefficient vector ***w***.

Step 6: Obtain the value of the point by calculating the weighted sum of the values and coefficients of the known points.

These are the steps for obtaining one unknown three-dimensional point. To obtain a smooth four-dimensional fingerprint map, steps 4 to 6 should be repeated.

### 2.2. Online Stage: PD Localization Using the Established Fingerprint Map

A PD can be localized using the RSSI values recorded by several sensors. In the first stage, the fingerprints of each sensor are established. Thus, the relevance of the RSSI values received by different sensors can be recorded according to the established fingerprint. In this paper, the relevance refers to the relative ratios of the RSSI values. When a standard PD power supply is tested at point m, the relative ratio of sensors *i* and *i* + 1 is

(26) $$\rho_{m}\left(i,i+1\right)=\frac{r_{i+1,m}^{\prime }}{r_{i,m}^{\prime }}$$

where $r_{i,m}^{\prime }$ and $r_{i+1,m}^{\prime }$ are the normalized RSSI values recorded by sensor *i* and *i*+1, respectively, during a discharge test at point *m*. The relative ratio matrix for point *m* is

(27) $$\rho_{m}=\left[\begin{array}{cc} \rho_{m}\left(1,2\right) & \ldots \;\rho_{m}\left(i,i+1\right) \end{array}\begin{array}{cc} \;\ldots & \rho_{m}\left(N-1,N\right) \end{array}\right]$$

When PD occurs, the RSSI values are also recorded by *N* sensors. Assuming that the position of the PD is *x,* the relative ratio matrix is

(28) $$\rho_{x}=\left[\begin{array}{cc} \rho_{x}\left(1,2\right) & \ldots \;\rho_{x}\left(i,i+1\right) \end{array}\begin{array}{cc} \;\ldots & \rho_{x}\left(N-1,N\right) \end{array}\right]$$

Although the RSSI values obtained by the sensors vary with the amplitude of the PD source, the relative ratios of the sensors remain stable. When a PD occurs, the local position can be converted to finding the point with the most similar relative ratio. The minimal deviation model can be written as

(29) min f=∑i=1N−1ρxi,i+1−ρmi,i+12 s.t. m∈P

where ***P*** is the set of points in the fingerprint. Several methods can be used to solve the model, such as exhaustive search, particle swarm optimization, or genetic algorithms. Since these algorithms are commonly used, they do not need to be repeated here.

**Figure 1:**
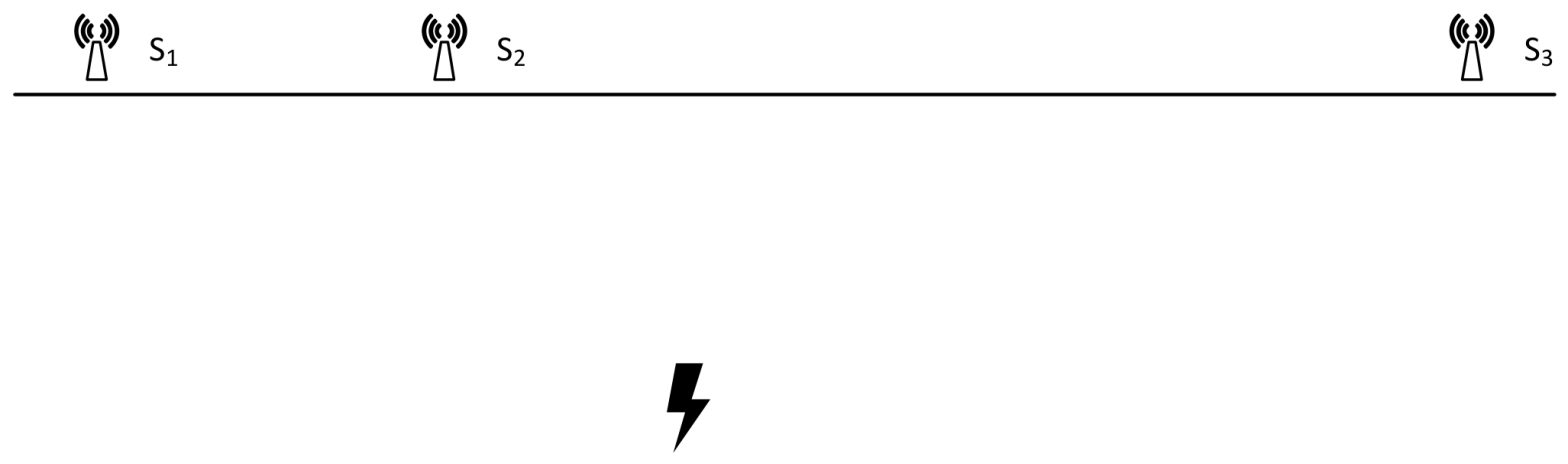
Layout diagram of the experiment.

In order to verify the relationship of the RSSI ratio, an experiment was conducted based on three sensors, as shown in Figure 1. The three sensors were arranged unevenly. A standard PD simulator discharged with varying intensity. The RSSI values of the three sensors are shown in Table 1. It can be seen from Table 1 that the coefficients of variation of the three relative ratios are 1.14%, 1.09% and 1.06%, respectively. The relative ratios between sensor RSSI values remain stable.

### 2.3. The Localization Process

**Figure 2:**
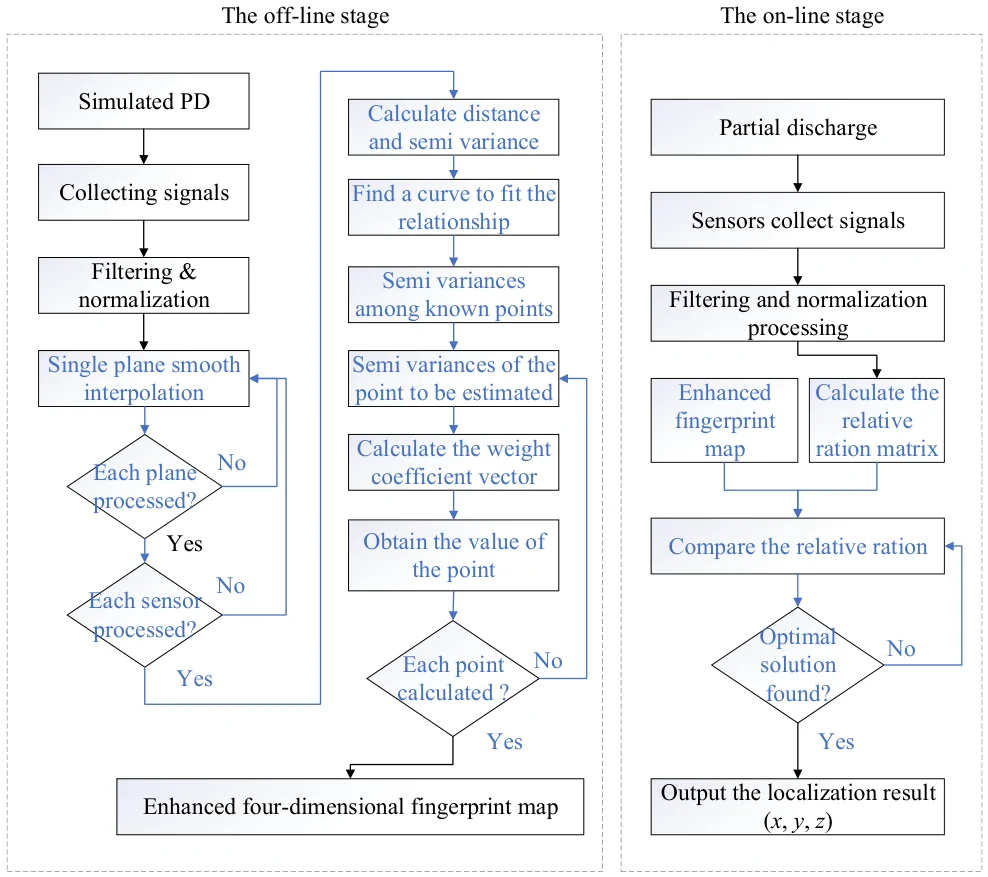
The PD localization process.

In this paper, the localization process consists of two stages, as shown in Figure 2. In the offline stage, partial discharges are first simulated at the test points of several planes. Interpolation is carried out for every plane. An enhanced four-dimensional fingerprint map for each sensor was then established. In the online stage, sensors collect the PD signals and the relative matrix is calculated. The position of the PD is finally localized by employing the minimal deviation model.

## 3. Experimental Verification

### 3.1. Experimental Scheme and Results

**Figure 3:**
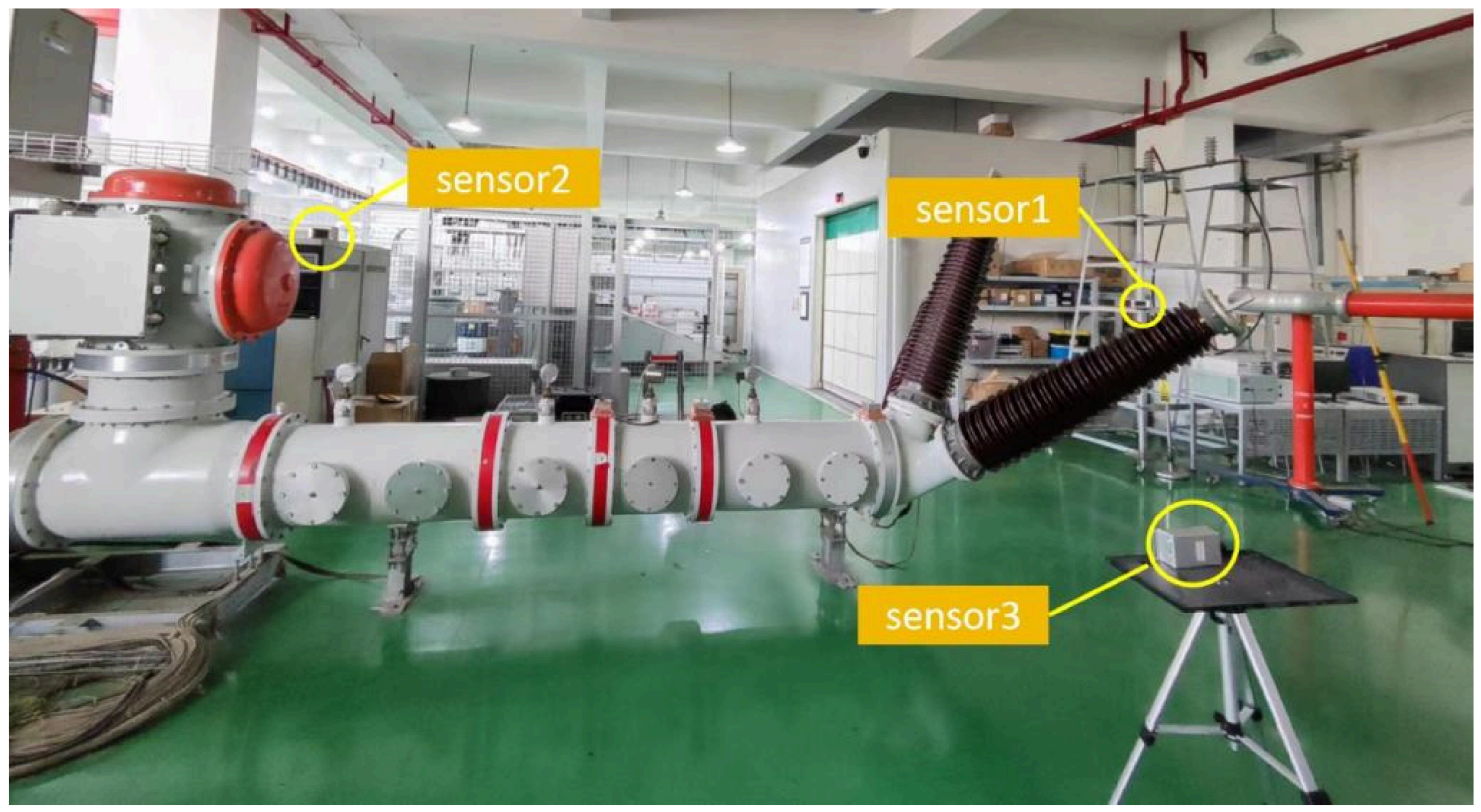
The test site used for verification.

The test site was located in a high-voltage test hall. The spatial environment was relatively complex, and therefore appropriate for simulating the substation environment. Figure 3 shows the test site, in which the transformer and other electrical equipment were not powered on and acted as obstacles. Three sensors were installed at the site, as shown in Figure 3.

**Figure 4:**
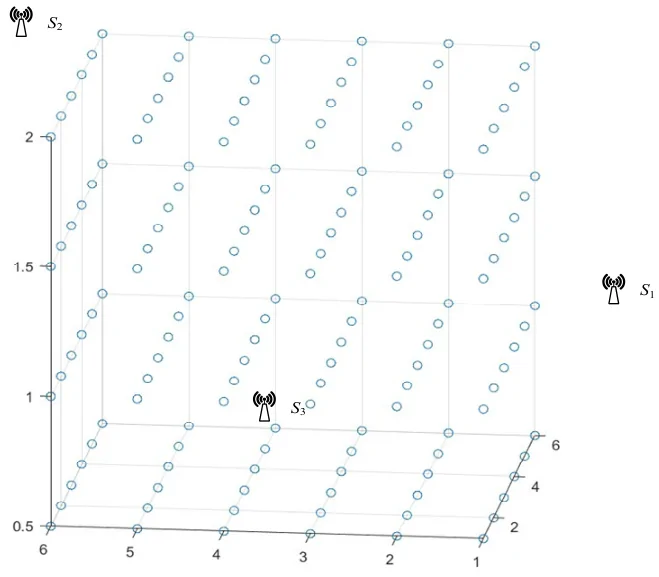
The distribution map of test points.

The test site was a 5 m × 5 m square area. Test points were set on planes with heights of 0.5 m, 1 m, 1.5 m and 2 m, as shown in Figure 4. On each plane, 36 test points, which are shown in Figure 4 as the blue dots were evenly distributed, and the distance between adjacent points was 1 m.

**Figure 5:**
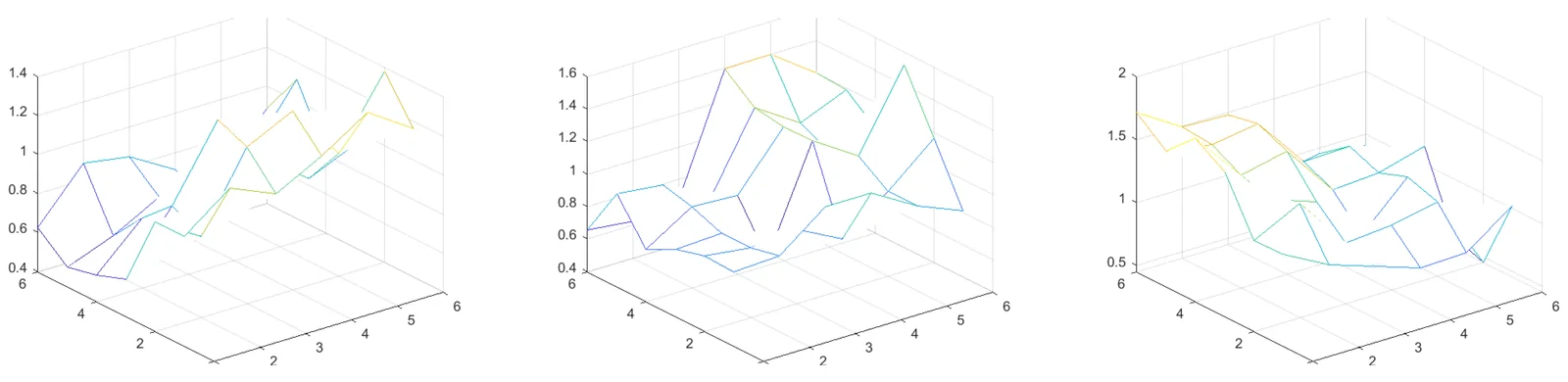
The normalized RSSI values of sensors 1 to 3 for test points on a 0.5 m high plane without single-plane smooth interpolation.

**Figure 6:**
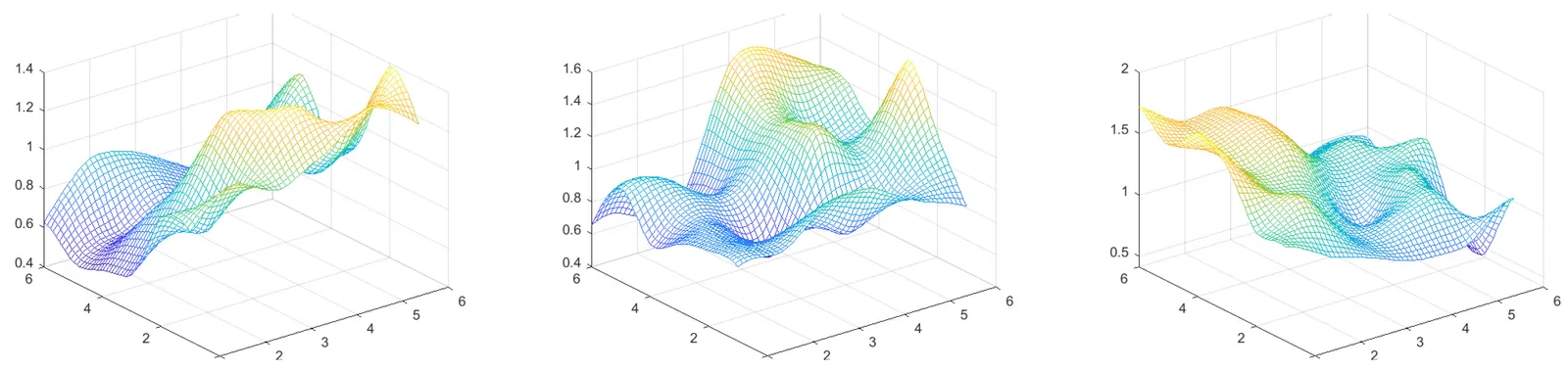
The normalized RSSI values of sensors 1 to 3 for test points on a 0.5 m high plane with single-plane smooth interpolation.

In the offline stage, the standard PD power supply was used to discharge at each test point on the planes, and all waveforms were recorded by the three sensors. After signal measurement and corresponding data processing, the normalized RSSI values were obtained. The results for the 0.5 m high plane both with and without single-plane smooth interpolation are shown in Figure 5 and Figure 6, respectively.

**Figure 7:**
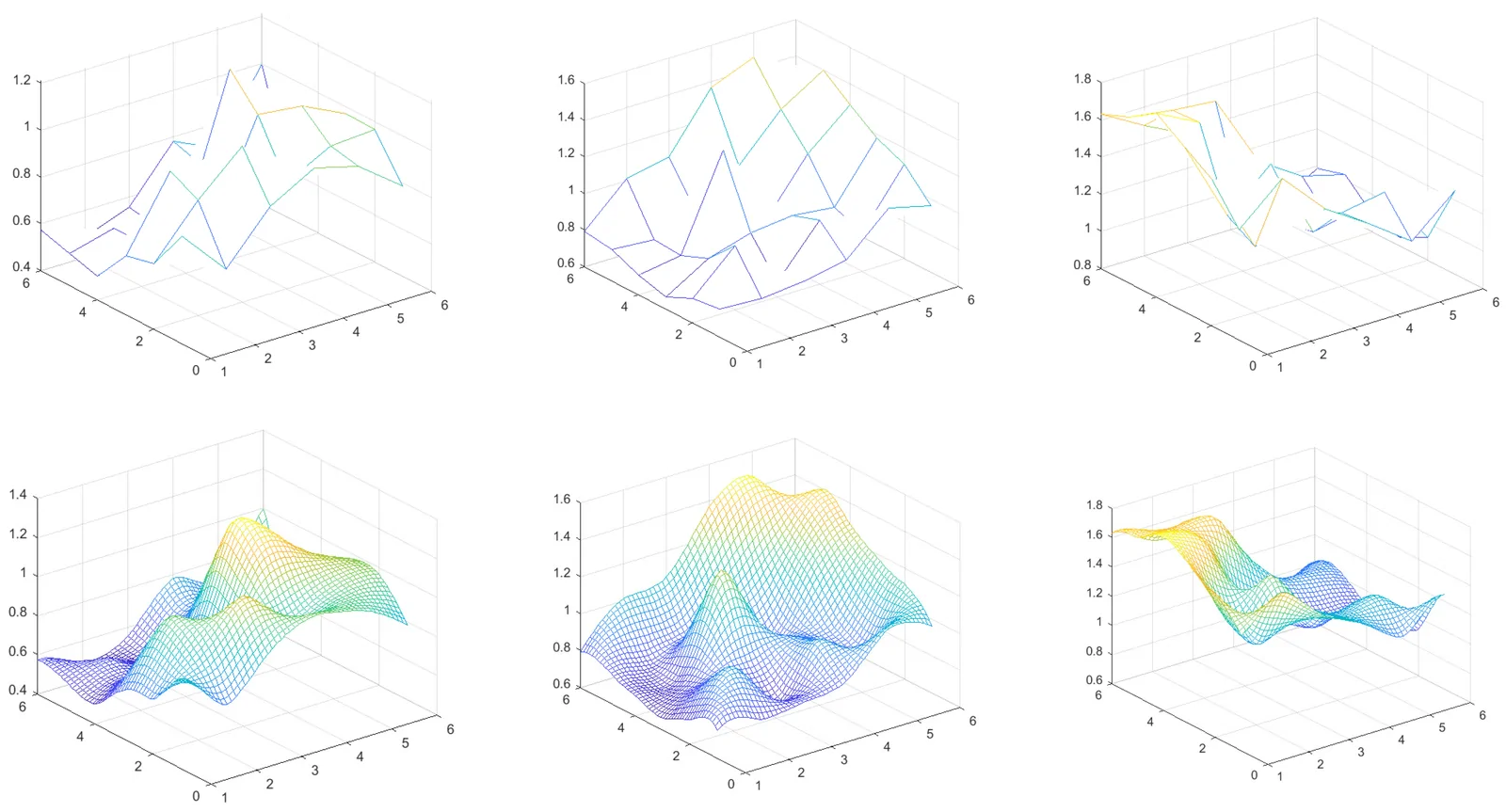
The normalized RSSI values of sensors 1 to 3 for test points on a 1 m high plane with single-plane smooth interpolation.

**Figure 8:**
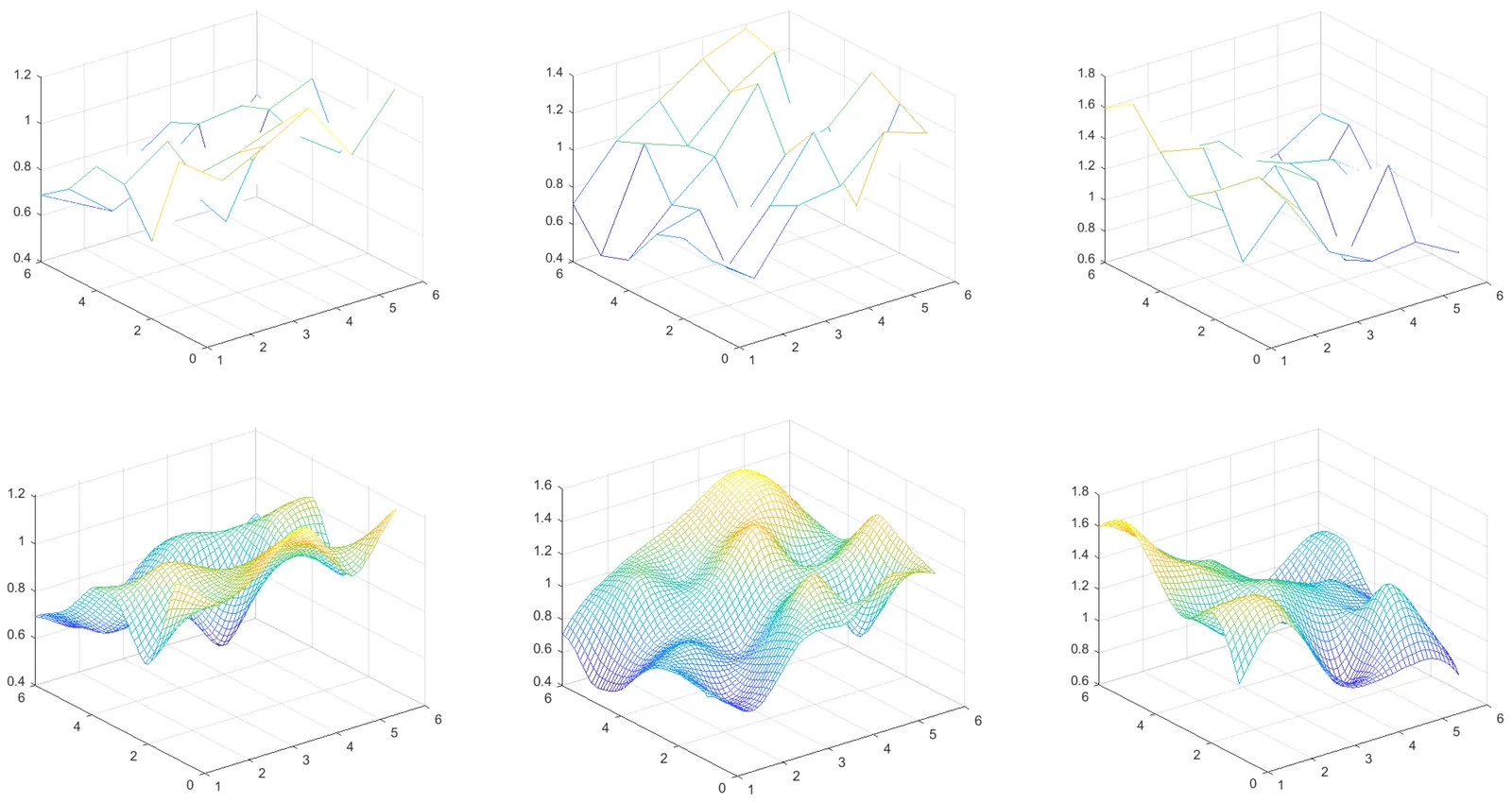
The normalized RSSI values of sensors 1 to 3 for test points on a 1.5 m high with and without single-plane smooth interpolation.

**Figure 9:**
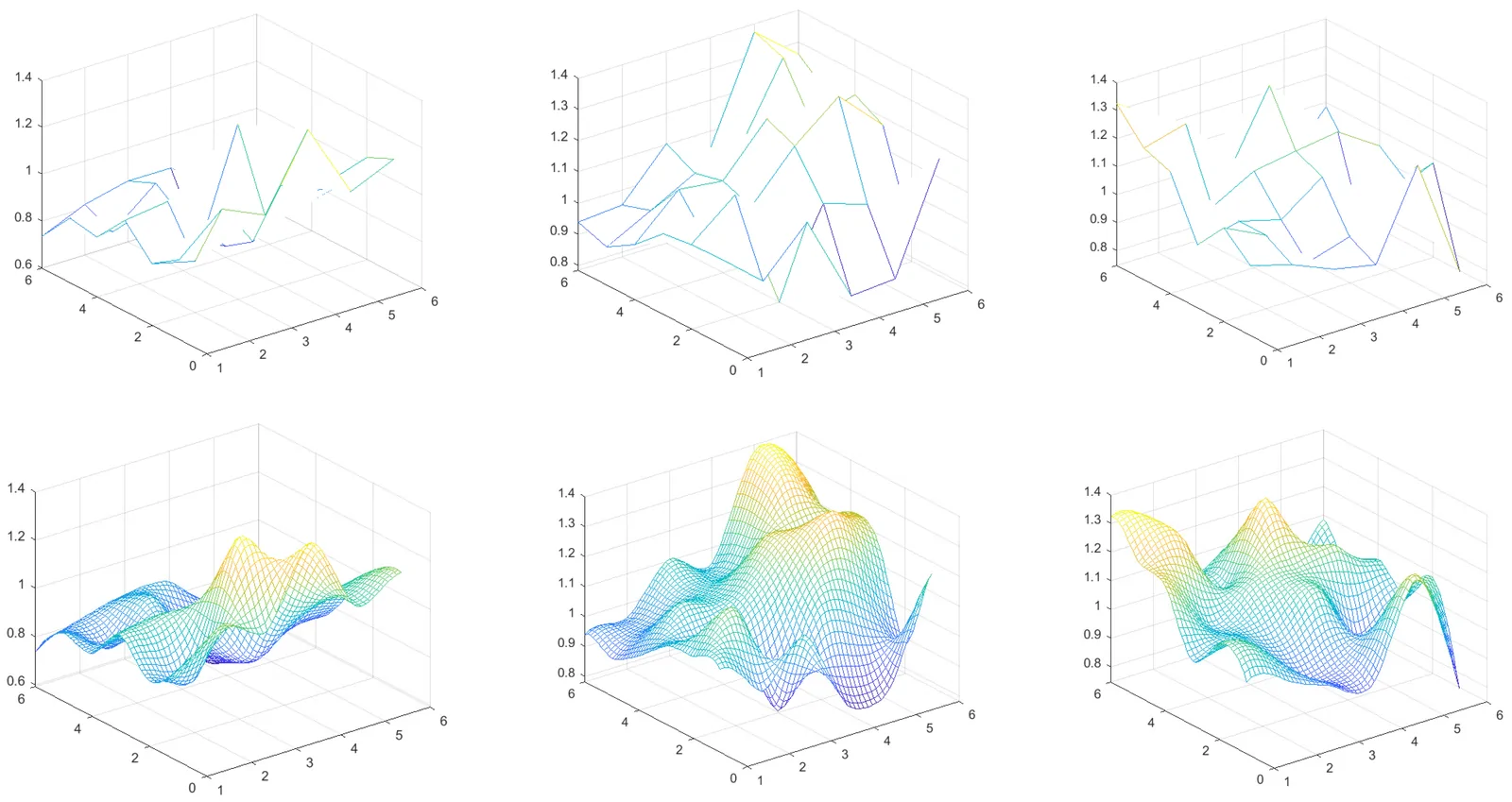
The normalized RSSI values of sensors 1 to 3 for test points on a 2 m high plane with and without single-plane smooth interpolation.

By comparing Figure 5 with Figure 6, it can be seen that when more values at other points on the plane were interpolated, the values transitioned more smoothly. The results from other planes with single-plane smooth interpolation are shown in Figure 7, Figure 8 and Figure 9.

To verify the effectiveness of the interpolation method, five measurement points from each plane were removed, and the RSSI values were reconstructed using biharmonic interpolation. The error results are shown in Table 2.

Similarly, five measurement points were removed from each plane, and the error results of the Kriging method are shown in Table 3.

Using the method described in Section 2.1.3, we estimated the RSSI values for three-dimensional positions at 0.1 m intervals. In order to select a suitable semivariogram model, a leave-one-out cross-validation method was employed to choose the model with the smallest CV-RMSE. The relationships between distance and semivariance for the three sensors are shown in Table 4.

**Figure 10:**
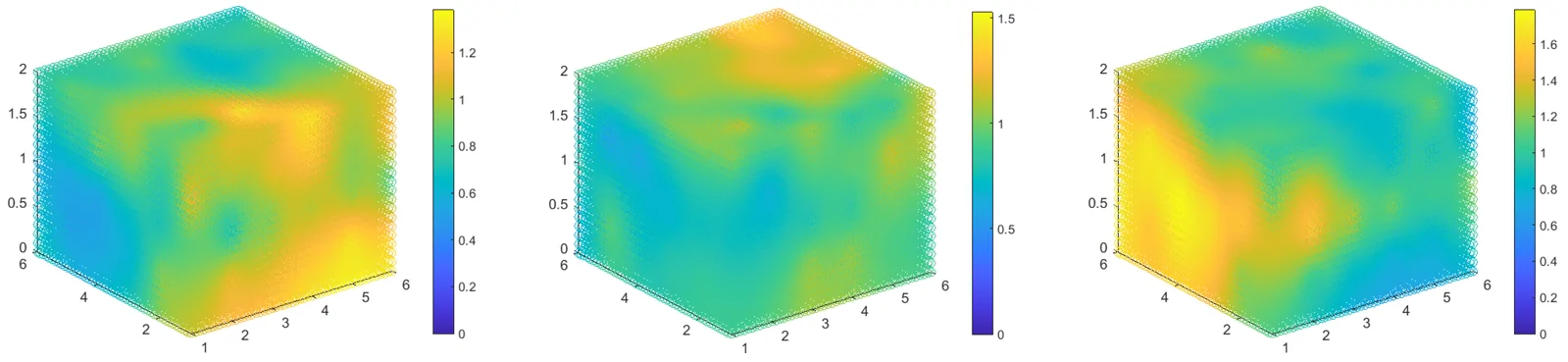
The enhanced fingerprint maps of sensors 1 to 3.

The enhanced fingerprint maps of three sensors are shown in Figure 10. It should be noted that the fingerprint maps have four dimensions, and the values for each three-dimensional point are shown by the colors in Figure 10.

It can be seen from Figure 10 that as the point gets closer to the sensor, the RSSI value becomes relatively larger, conforming to the attenuation trend of signal propagation. The fingerprint maps can accurately reflect the spatial information of the test site. The irregularity of the fingerprint maps is mainly caused by the complex spatial environment of the test site.

**Figure 11:**
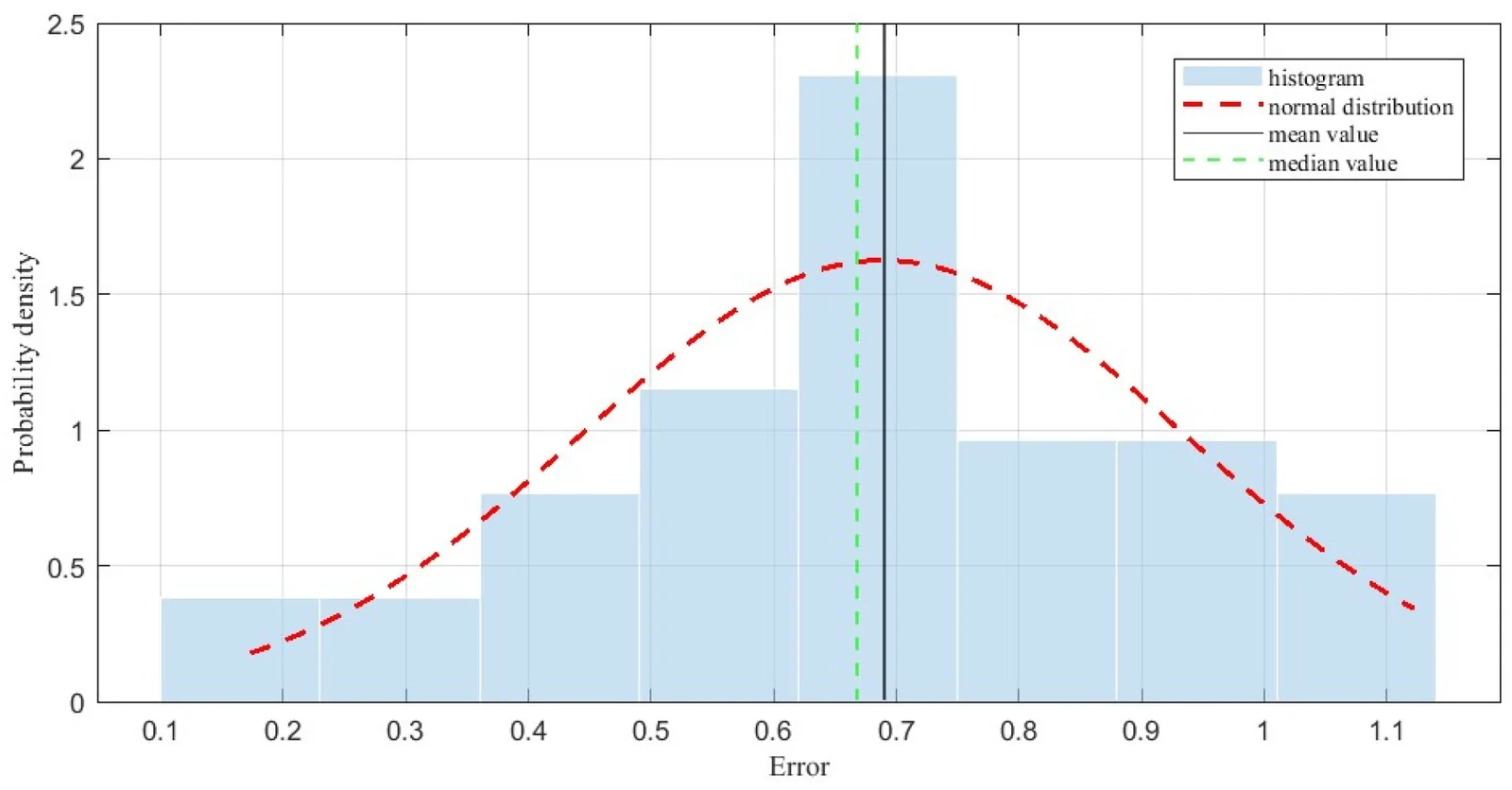
Localization error distribution histogram.

In the second stage, in order to test the accuracy of the proposed method, the PD power supply discharged at 40 three-dimensional points equally distributed among planes with heights of 0.3 m, 0.8 m, 1.3 m and 1.8 m. The RSSI values of each sensor were also recorded, and an exhaustive algorithm was employed to solve the minimal deviation model. The localization error distribution histogram is shown in Figure 11; the standard deviation was 0.25 m, the average and median errors were 0.69 m and 0.67 m, respectively, and the 95% confidence interval of the mean error was [0.61 m, 0.77 m].

The high-voltage power equipment in substations is generally large in size, and there is also a certain electrical safety distance between the equipment of different phases. A distance of 0.69 m is sufficient for the PD localization in the majority of devices. Certain difficulties remain for some small-sized devices and for establishing the specific location of the PD on the device. However, the location range can be determined based on the localization result, and, combined with a handheld detection tool at close range, the exact PD location can be easily obtained.

Another possible method might be to employ a pattern recognition algorithm, such as a BP (backpropagation) neural network, to achieve three-dimensional localization. In this approach, the RSSI values collected from the test points are used to train a BP neural network. Then, the trained BP neural network can be used to localize PD based on the RSSI values detected when a PD occurs. This task is treated as a regression problem. The values obtained from the three sensors corresponding to each test point were used as input feature data, and the three-dimensional coordinates of each test point were used as output data. A normalization method was used for data preprocessing. The input layer, hidden layer and output layer had three, ten, and three neurons, respectively. The hidden layer used the hyperbolic tangent sigmoid function, and the output layer used a linear function. The training function adopted the Levenberg–Marquardt backpropagation algorithm, with a maximum of 1000 training epochs, a target error of 1 × 10^−5^ and a minimum gradient of 1 × 10^−7^. The data were randomly divided into a 70% training set, a 15% validation set and a 15% internal test set. The number of repeated training runs was 10. The localization results of the BP neural network method are shown in Table 5.

It can be seen from Table 5 that the average error of the proposed method is 0.69 m, which is much better than that of the BP neural network method. The minimum and maximum errors are 0.17 m and 1.12 m, respectively; therefore, the proposed method shows better overall performance.

### 3.2. Effects of the Objective of the Minimal Deviation Model

In this paper, the relative ratios of sensors were used to formulate the objective of the minimal deviation model. The key formula is Equation (26), which determines the composition of the objective function. To analyze the effects of the objective, other calculation formulae, such as the sum, difference, and product of the sensors, were used. Three different forms of Equation (26) are shown below:

(30) $$\mathrm{form}2\;\rho_{m}\left(i,i+1\right)=r_{i+1,m}^{\prime }+r_{i,m}^{\prime }$$

(31) $$\mathrm{form}3\;\rho_{m}\left(i,i+1\right)=r_{i+1,m}^{\prime }-r_{i,m}^{\prime }$$

(32) $$\mathrm{form}4\;\rho_{m}\left(i,i+1\right)=r_{i+1,m}^{\prime }\times \;r_{i,m}^{\prime }$$

The localization results of different objectives are shown in Table 6. It can be seen from Table 6 that the average errors are on the same order of magnitude. The underlying logic of the objective proposed in this paper is to find the point where the relative relationship between the values received by each sensor is the most consistent. Since $r_{i,m}^{\prime }$ is normalized with the means of the values of three sensors, $r_{i,m}^{\prime }$ itself carries a certain amount of relative relationship information regarding the three sensors. Various forms of objectives based on $r_{i,m}^{\prime }$ meet the same underlying logic, so results on the same order of magnitude can be obtained.

### 3.3. Effects of the Number of Testing Points

**Figure 12:**
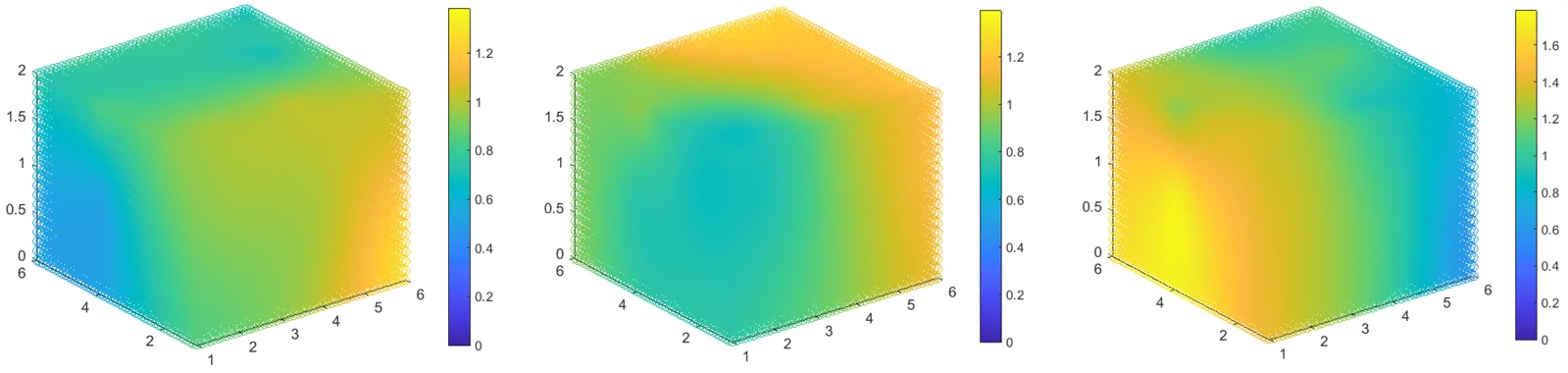
The fingerprint maps of sensors 1 to 3 with 18 test points.

The fingerprint maps were established based on the data of a few test points. In order to analyze the effect of the number of test points on the results of PD localization, different numbers were tested. For example, if one test point was left out every eight intervals, the total number of test points was reduced from 144 to 18. The established fingerprint maps are shown in Figure 12.

**Figure 13:**
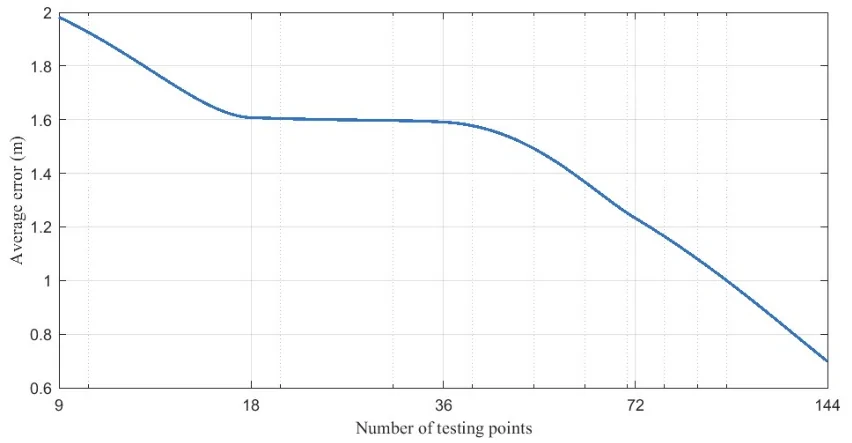
The accuracy-versus-number-of-calibration-points curve.

The accuracy-versus-number-of-calibration-points curve is shown in Figure 13. The average error decreases as the number of test points increases. It can be seen from the figure that, to achieve a positioning accuracy of approximately 1 m, the minimum number of calibration points should be 100. There is a clear difference between Figure 10 and Figure 12: Figure 12 lost many features due to having fewer test points. Thus, the difference between the established fingerprint maps and the actual maps is magnified, and the average error increases as the number of test points decreases. The relationship between the calibration workload, number of measurement points, and positioning error is shown in Table 7.

## 4. Conclusions

This paper presents a three-dimensional localization method based on RSSI fingerprint maps. To obtain a detailed, accurate four-dimensional fingerprint map with only a few three-dimensional test points, a smooth interpolation method was first employed to address data augmentation issues on a single plane. Then, the Kriging method was used to assist in the creation of a four-dimensional fingerprint map across planes. Finally, a minimal deviation model was proposed to locate PD using the established four-dimensional fingerprint map. The contributions of the proposed method are described as follows.

Firstly, a novel three-dimensional localization method was proposed based on the creation of an RSSI fingerprint map. The average error of the proposed method is 0.69 m, superior to that of the BP neural network method, another possible three-dimensional method. This level of accuracy is sufficient to locate PD in equipment. The proposed PD localization method is environmentally adaptable and highly accurate, making it suitable for widespread use.

Secondly, the underlying logic of the objective proposed in this paper is that the relative relationship between the RSSI values of the sensors at each three-dimensional point is relatively stable. Thus, finding the optimal point on the map means finding the point with the most consistent relative ratio of the values of the sensors. Objectives formulated based on this logic can yield good localization results.

Thirdly, the average error increases as the number of test points decreases. Thus, it is necessary to comprehensively consider the feasibility of the operation and the consumption of resources to select the appropriate quantity of test points that achieves sufficient accuracy.

## Figures and Tables

**Table 1:** The RSSI values of three sensors.

Test Serial Number	S_1_	S_2_	S_3_	S_1_/S_3_	S_2_/S_1_	S_3_/S_2_
1	0.985	1.203	0.811	1.215	1.221	0.674
2	0.974	1.213	0.814	1.197	1.246	0.671
3	0.961	1.223	0.816	1.178	1.272	0.667
4	0.972	1.219	0.809	1.201	1.255	0.664
5	0.982	1.216	0.803	1.223	1.238	0.660
6	0.976	1.211	0.813	1.200	1.241	0.671
7	0.970	1.206	0.824	1.177	1.244	0.683
8	0.972	1.210	0.818	1.189	1.244	0.676
9	0.975	1.214	0.811	1.202	1.245	0.668
10	0.980	1.200	0.820	1.195	1.225	0.683
coefficient of variation				1.14%	1.09%	1.06%

**Table 2:** The error results of biharmonic interpolation.

Plans	RMSE	MAE	R^2^
0.5 m	0.091	0.070	0.880
1.0 m	0.082	0.069	0.889
1.5 m	0.079	0.061	0.866
2.0 m	0.063	0.055	0.869

**Table 3:** The error results of the Kriging method.

Plans	RMSE	MAE	R^2^
0.5 m	0.117	0.101	0.859
1.0 m	0.090	0.071	0.898
1.5 m	0.089	0.079	0.851
2.0 m	0.074	0.061	0.855

**Table 4:** Relationships between distance and semivariance for the three sensors.

Parameters	Sensor 1	Sensor 2	Sensor 3
Optimal semivariogram model	spherical	spherical	spherical
Nugget	0	0.0005	0
Sill	0.0437	0.0459	0.0975
Range	4.5690	5.1054	7.2935
Fitting algorithm	least squares	least squares	least squares
Neighborhood size	global	global	global
Anisotropy assumption	isotropic	isotropic	isotropic
CV-RMSE (spherical)	0.0216	0.0217	0.0230
CV-RMSE (exponential)	0.0217	0.0219	0.0231
CV-RMSE (gaussian)	0.1189	0.6962	0.2645
CV-RMSE (power)	0.0239	0.0247	0.0237

**Table 5:** Comparisons of different methods.

Method	Average Error (m)	Minimum Error (m)	Maximum Error (m)
Proposed method	0.69	0.17	1.12
BP neural network	2.14	0.58	5.35

**Table 6:** Comparisons for different formulas of Equation (26).

Method	Proposed Method	Form 2	Form 3	Form 4
Average error(m)	0.69	0.89	0.95	0.97

**Table 7:** The relationship between the calibration workload, number of measurement points and positioning error.

Number of Measurement Points	Calibration Workload (h)	Positioning Error (m)
144	4.8	0.69
72	2.4	1.23
36	1.2	1.59
18	0.6	1.61
9	0.3	1.98

## Data Availability

The data presented in this study are available from the corresponding author upon reasonable request.
